# Clinical Characteristics and Outcomes Until 2 Years of Age in Preterm Infants With Typical Chest Imaging Findings of Bronchopulmonary Dysplasia: A Propensity Score Analysis

**DOI:** 10.3389/fped.2021.712516

**Published:** 2021-08-23

**Authors:** Qiqi Ruan, Jianhui Wang, Yuan Shi

**Affiliations:** ^1^Department of Neonatology, Children's Hospital of Chongqing Medical University, Chongqing, China; ^2^National Clinical Research Center for Child Health and Disorders, Chongqing, China; ^3^Ministry of Education Key Laboratory of Child Development and Disorders, Chongqing, China; ^4^Chongqing Key Laboratory of Pediatrics, Chongqing, China

**Keywords:** bronchopulmonary dysplasia, typical imaging findings, clinical characteristics, outcomes, preterm infants, propensity score

## Abstract

**Objective:** The goal of the current study was to assess the associations of typical chest imaging findings of bronchopulmonary dysplasia (BPD) in preterm infants with clinical characteristics and outcomes until 2 years of age.

**Method:** This retrospective cohort study enrolled 256 preterm infants with BPD who were admitted between 2014 and 2018. A propensity score analysis was used to adjust for confounding factors. The primary outcomes were the severity of BPD, home oxygen therapy (HOT) at discharge and mortality between 28 days after birth and 2 years of age. A multivariate logistic regression analysis was performed to identify related variables of mortality.

**Results:** Seventy-eight patients with typical chest imaging findings were enrolled, of which 50 (64.1%) were first found by CXR, while 28 (35.9%) were first found by CT. In addition, 85.9% (67/78) were discovered before 36 weeks postmenstrual age (PMA) (gestational age [GA] < 32 weeks) or before 56 days after birth (GA > 32 weeks). After propensity score matching, the matched groups consisted of 58 pairs of patients. Those with typical imaging findings had a remarkably higher mortality rate (29.3 vs. 12.1%, *p* = 0.022, OR 3.021), higher proportion of severe BPD (32.8 vs. 12.1%, *p* = 0.003, OR 4.669) and higher rate of HOT at discharge (74.1 vs. 46.6%, *p* = 0.002, OR 3.291) than those without typical imaging findings. The multivariate logistic regression analysis showed that typical imaging findings ≤ 7 days and typical typical imaging findings >7 days were independent risk factors for mortality in preterm infants with BPD (OR 7.794, *p* = 0.004; OR 4.533, *p* = 0.001).

**Conclusions:** More attention should be given to chest imaging findings of BPD, especially in the early stage (within 7 days). Early recognition of the development of BPD helps early individualized treatment of BPD.

**Clinical Trial Registration:**www.ClinicalTrials.gov, identifier: NCT04163822.

## Introduction

Bronchopulmonary dysplasia (BPD) is a common chronic respiratory disease in preterm infants (1). The increase in the survival rate of premature babies following the improvement of perinatal treatment and care has caused an increase in the incidence of BPD in recent years (2), which has seriously affected the quality of life of preterm infants. According to the consensus reached at the workshop sponsored by the National Institute of Child Health and Human Development (NICHD) in 2001 (3), BPD was clinically defined based on oxygen dependency in preterm infants. However, the refined NICHD definition of BPD in 2018 (4) emphasized imaging findings to support a diagnosis of lung parenchyma disease.

Fibrotic opacities and cystic changes on chest imaging [chest X-ray (CXR) or computed tomography (CT) scan] were considered typical findings in BPD patients (5–7). In patients with severe BPD, the presence of bubbles/cystic appearance on CXR after 28 days of life was reported to be an important factor, and typical imaging findings can predict a poor pulmonary outcome in BPD patients (8). BPD is associated with poor outcomes (9). Although many studies have been conducted on BPD, there are limited reports specifically evaluating the association of typical imaging findings with clinical characteristics and later outcomes in patients with BPD.

We hypothesized that BPD with typical imaging findings was likely to be a particular subgroup of this entity, with a unique etiology, clinical characteristics and prognosis. Therefore, this retrospective study aimed to compare clinical characteristics, short-term outcomes and follow-up data until 2 years of age in preterm infants with or without typical imaging findings of BPD on CXR or CT scan during the entire hospital stay. A propensity score analysis was used to reduce bias between the two groups, and multivariate logistic regression analysis was performed to identify factors related to mortality in preterm infants with BPD.

## Methods

### Definitions of the Relevant Concepts

BPD was diagnosed and graded according to the 2001 NICHD consensus (3). Oxygen dependence (10) was defined as requiring oxygen supplementation for more than 12 h a day to maintain oxygen saturation ≥92%. Home oxygen therapy (HOT) at discharge (11) was used to maintain oxygen saturation at ≥95% by using short-term oxygen rather than continuous supplemental oxygen.

Typical chest imaging findings include fibrotic opacities and cysts (7, 12, 13) on CXR or CT scans during the entire hospital stay. Fibrotic opacities (14) were defined as an area with diffused and reticular density or triangular subpleural opacities, and cystic appearance (8) was defined as a lucent parenchymal area with a clear boundary. Chorioamnionitis (CAM) was diagnosed based on clinical features, such as maternal fever, localized pain and leukocytosis during pregnancy and delivery. Prenatal glucocorticoid administration was defined as the administration of at least one maternal dose of glucocorticoids between 24 h and 7 days prior to delivery. The meta-analysis by Shah et al. (15) showed that there was no significant difference between infants who received inhaled and systemic steroids in the incidence of death or BPD at 36 weeks PMA. Then, postnatal glucocorticoid administration was defined as the administration of inhaled or systemic glucocorticoids to preterm infants at any time after delivery. Repeated surfactant dose (RSD) (16) was defined as supplementation with at least two doses of pulmonary surfactant (PS).

Sepsis was diagnosed following the Chinese Expert Consensus (version 2019) (17). Patent ductus arteriosus (PDA) was diagnosed by echocardiography if there was a significant left-to-right shunt through the ductus arteriosus. Intraventricular hemorrhage (18) (IVH) was defined according to Papile's classification (stage III to IV). Necrotizing enterocolitis (NEC) (19) was defined as level II or higher according to Bell's classification. Stunting was defined as >2 standard deviations (SD) below the mean length for age, and underweight was defined as >2 SD below the mean weight for age. Weight and length were calculated with Chinese growth reference standards (20). Wheezing disorders were defined as a physician diagnosis of wheezing exposure treated with anti-asthma drugs (bronchodilators and corticosteroids) (21).

### Patients

We performed a retrospective cohort study using data from the Department of Neonatology, Children's Hospital of Chongqing Medical University (CHCMU), and the patients were followed by telephone. This study was approved by the Institutional Review Board of CHCMU (2019-209) and was registered on Clinicaltrials.gov (NCT04163822). Eligible patients fulfilled the following three criteria simultaneously: (1) BPD diagnosis according to the 2001 NICHD consensus; (2) chest imaging examination (CXR or CT) in the first week after birth; and (3) hospitalization within the first 7 days after birth.

Patients were excluded from the study if they met one of the following conditions: (1) major congenital malformations or laboratory-confirmed chromosomal abnormalities; (2) inadequate clinical data or missing chest imaging data; or (3) loss to follow-up.

### Data Collection

Relevant data were retrospectively collected, including demographics [i.e., sex, gestational age, age at admission, birth weight (BW)], perinatal factors [i.e., pregnancy-induced hypertension (PIH), CAM, cesarean section, prenatal glucocorticoid administration, respiratory distress syndrome (RDS), sepsis, PDA, 5-min Apgar score], management during hospitalization [i.e., surfactant administration, noninvasive and invasive mechanical ventilation (IMV), postnatal glucocorticoid administration, packed red blood cell (PRBC) transfusion], complications during hospitalization [i.e., IVH, retinopathy of prematurity (ROP), NEC], and outcomes.

Primary outcomes were the severity of BPD, HOT at discharge, and mortality between 28 days after birth and 2 years of age. Secondary outcomes were (1) duration of hospital stay; (2) duration of oxygen supplementation; (3) routine physical assessment; (4) wheezing disorders; and (5) clinical visits and rehospitalizations for a respiratory reason between discharge and follow-up. The long-term outcomes were evaluated until 2 years of age.

### Statistical Analysis

Statistical analysis was performed using SPSS 26.0. We used the Shapiro-Wilk test to analyze the normality of the data distribution. Variables without normal distributions were analyzed using the Wilcoxon-Mann-Whitney test and are reported as medians [interquartile ranges (IQRs)]. All normally distributed variables were analyzed using a *t*-test for comparisons of two independent groups and are presented as the means ± standard deviations. Categorical variables were analyzed using the chi-square test or Fisher's exact tests and are reported as the numbers (%) of subjects.

To further evaluate the associations between typical chest imaging findings and the primary and secondary outcomes, a propensity score analysis with 1:1 matching was performed. The following covariates were included based on previous studies (22–26): GA, BW, cesarean section, CAM, PIH, sepsis, RDS, PDA, prenatal glucocorticoid administration, postnatal glucocorticoid administration, NEC, IVH, RSD, IMV ≥ 7 days, ≥2 PRBC transfusions. The nearest neighbor matching method was used to select each matched pair by using calipers with a width equal to 0.01 of the standard deviation of the logit of the propensity score. Typical chest imaging findings and the above 15 covariates were analyzed for collinearity and incorporated into the multivariate binary regression model, and the forward likelihood ratio (LR) was used to select the variables to further determine the independent factors of BPD infant mortality. A *p*-value < 0.05 was considered statistically significant.

## Results

### Subjects

**Figure 1:**
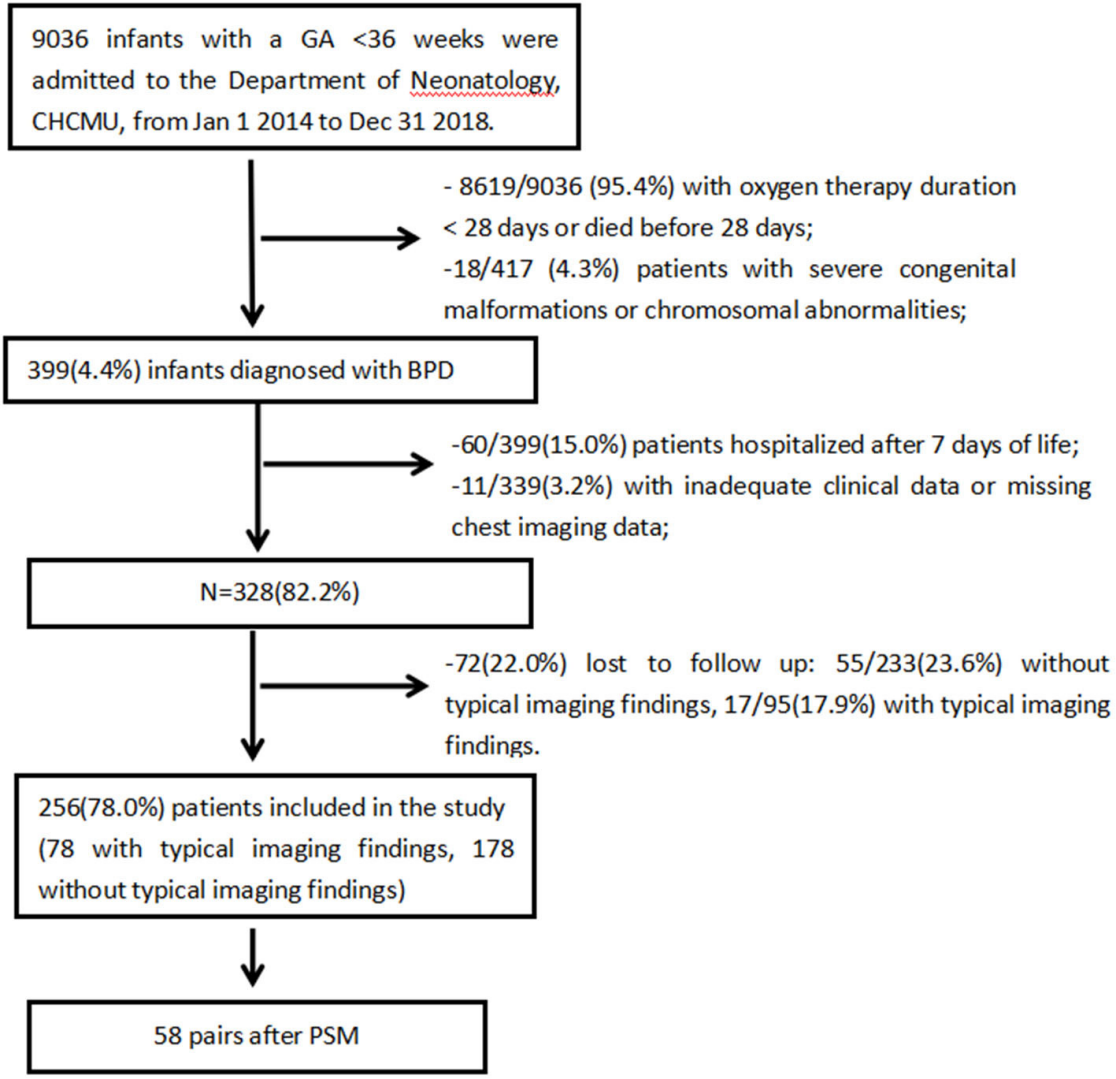
Flowchart of the inclusion process.

The inclusion process is shown in Figure 1. A total of 9036 preterm infants with GA <36 weeks were admitted to the Department of Neonatology, CHCMU, from January 1, 2014, to December 31, 2018. Among them, 399 (4.4%) infants were diagnosed with BPD. Then, infants who were hospitalized after 7 days of life with missing data and lost to follow-up were excluded. There was no significant difference in baseline characteristics between the included infants and those lost to follow-up, and there was no significant difference in the loss to follow-up rate between the two groups (17.9% (17/95) vs. 23.6% (55/233), *p* = 0.237). This showed that the loss to follow-up was random and would not affect outcomes significantly.

Ultimately, 256 preterm infants were enrolled; 78 (30.5%) had typical chest imaging findings, whereas 178 (69.5%) did not have typical imaging findings. Propensity score matching (PSM) systematically excluded infants who lacked a control group: those had lower GA, higher rates of sepsis, IMV ≥ 7 days and PRBC transfusion ≥2 times. After PSM, the matched groups consisted of 58 pairs of patients.

### Information of Typical Imaging Findings

**Figure 2:**
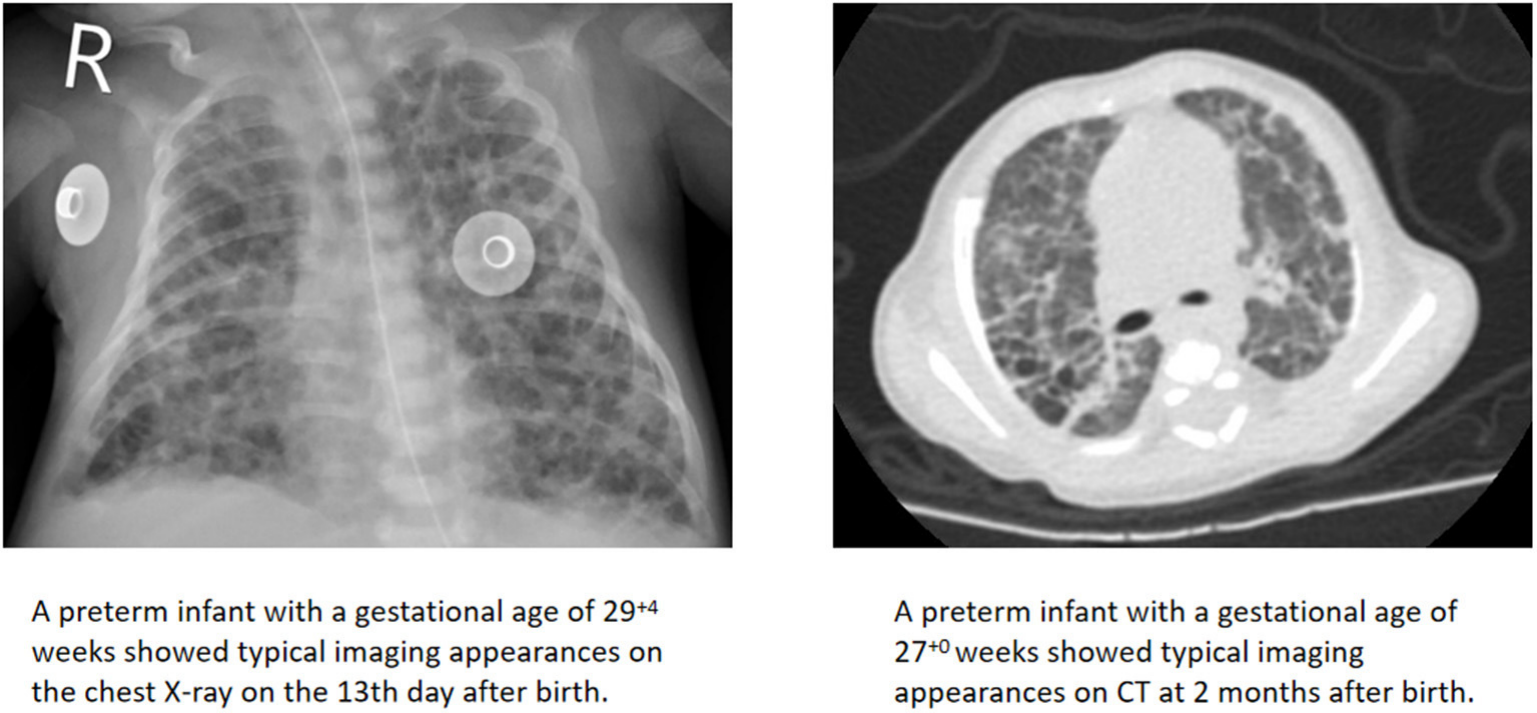
Representative of typical imaging findings.

A total of 78 infants had typical imaging findings, of which 50 (64.1%) were first found by CXR, while 28 (35.9%) were first found by CT. In our unit, infants in the neonatal intensive care units (NICUs) had a chest radiograph at the bedside. CT scans required sedation and had the risk of respiratory depression, so they were performed when the child's condition was relatively stable. All chest imaging appearances were jointly assessed by two radiologists to identify whether the typical patterns of BPD were present (Figure 2). If there were disagreements, the decision was made through internal discussion in the department before the clinical report was issued.

**Figure 3:**
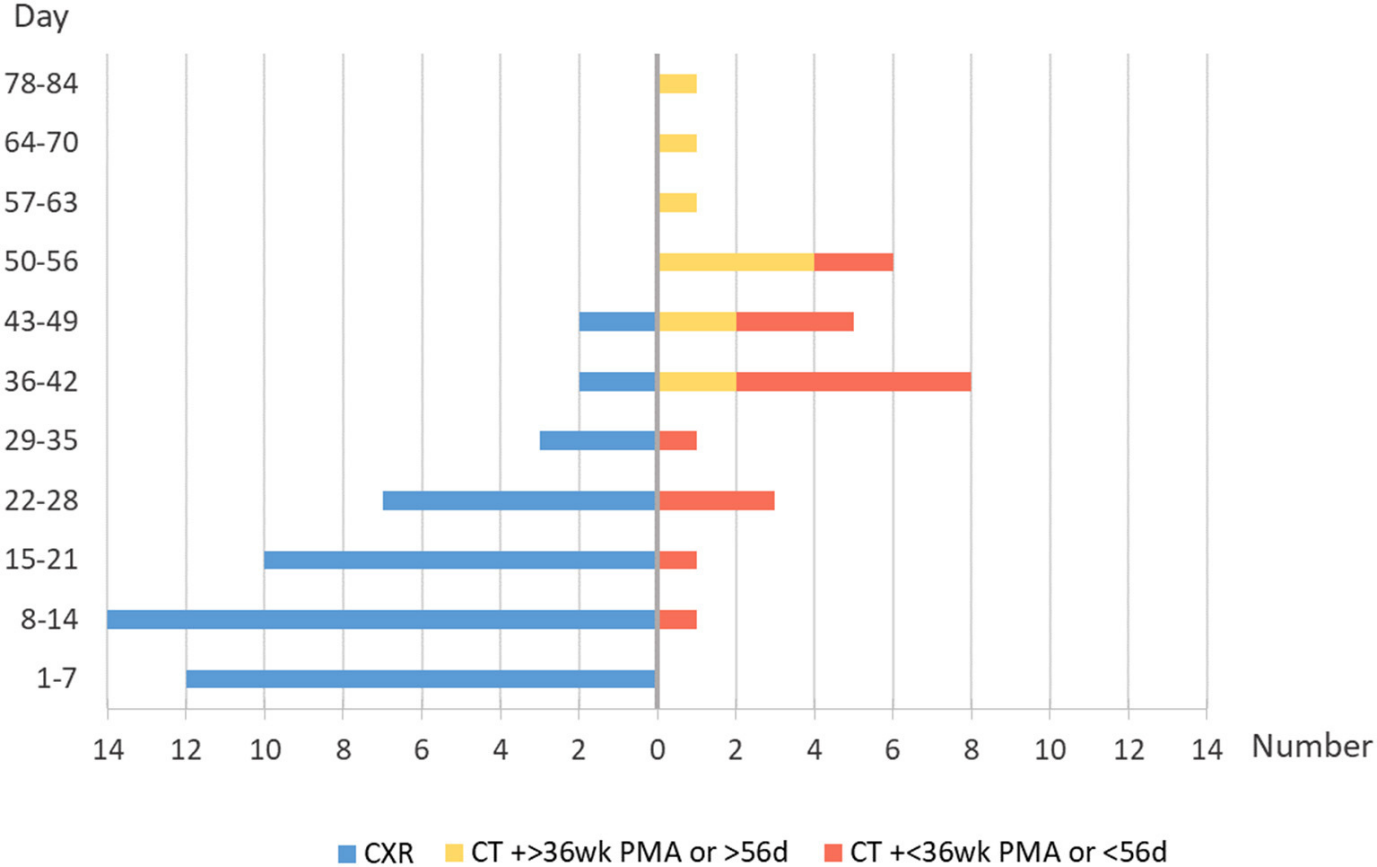
Timing and modalities of the typical imaging appearance first showed.

This was a retrospective study without intervention and blinding, but radiologists assessing the imaging appearances were basically unaware of the outcomes of interest. As shown in Figure 3, 15.4% (12/78) of the typical imaging patterns were first discovered within 7 days of life, and 57.7% (45/78) were found before 28 days of life, when the diagnostic criteria for BPD had not been met. In addition, 85.9% (67/78) were found before 36 weeks postmenstrual age (PMA) (GA <32 weeks) or before 56 days after birth (GA > 32 weeks), when the severity of BPD was not evaluated. Thus, the radiologists were not aware of the primary outcomes. Only 14.1% (11/78) of the typical patterns were discovered after clinical evaluation of the severity of BPD; not surprisingly, these 11 cases were all CT scans. Radiologists may know the diagnosis, but they were unaware of the mortality. The review of CT appearances was more objective and detailed than CXR.

### Clinical Characteristics of BPD Infants

The patient characteristics of the two groups are shown in Table 1. Fifteen covariates were included in the propensity score-matched model. Before matching was implemented, seven out of the 15 covariates were significantly different between the two groups.

**Table 1:** Characteristics of the preterm infants included in the propensity score analysis.

	**Before matching**			**After matching**		
	**Typical imaging findings (−) (*n* = 178)**	**Typical imaging findings (+) (*n* = 78)**	***P* value**	**Typical imaging findings (−) (*n* = 58)**	**Typical imaging findings (+) (*n* = 58)**	***p* value**
GA (wk)	30.6 (29.3, 32.1)	29.4 (28, 31.1)	0.001	29.3 (28.1, 30.6)	29.4 (28, 32.3)	0.327
BW (g)	1400 (1220, 1650)	1225 (1017.5, 1555)	0.001	1300 (1100, 1485)	1280 (1041.2, 1580)	0.985
Cesarean section	104 (58.4)	41 (52.6)	0.384	27 (46.6)	32 (55.2)	0.353
Prenatal glucocorticoid administration	89 (50.0)	43 (55.1)	0.450	27 (46.6)	29 (50.0)	0.710
PIH	30 (16.9)	13 (16.7)	0.971	8 (13.8)	9 (15.5)	0.793
CAM	7 (3.9)	5 (6.4)	0.388	2 (2.4)	1 (1.7)	1.000
RDS	130 (73.0)	66 (84.6)	0.044	47 (81.0)	48 (82.8)	0.809
Sepsis	110 (61.8)	59 (75.6)	0.031	44 (75.9)	41 (70.7)	0.529
PDA	119 (66.9)	60 (76.9)	0.106	44 (75.9)	44 (75.9)	1.000
Postnatal glucocorticoid administration	117 (65.7)	63 (80.8)	0.015	40 (69.0)	44 (75.9)	0.406
RSD	49 (27.5)	24 (30.8)	0.597	14 (24.1)	16 (27.6)	0.672
IMV ≥ 7 days	73 (41.0)	53 (67.9)	<0.001	30 (51.7)	33 (56.9)	0.576
PRBC transfusion ≥ 2	55 (30.9)	36 (46.2)	0.019	25 (43.1)	23 (39.7)	0.706
IVH	8 (4.5)	5 (6.4)	0.939	3 (5.2)	2 (3.4)	1.000
NEC	8 (4.5)	1 (1.3)	0.360	2 (3.4)	1 (1.7)	1.000

*Data are presented as medians (IQRs) or numbers (%). GA, Gestational age; BW, Birth weight; PIH, Pregnancy-induced hypertension; CAM, Chorioamnionitis; RDS, Respiratory distress syndrome; PDA, Patent ductus arteriosus; RSD, Repeated surfactant dose; IMV, Invasive mechanical ventilation; PRBC, Packed red blood cell transfusion; IVH, Intraventricular hemorrhage; NEC, Necrotizing enterocolitis*.

The typical imaging finding (+) group had a lower GA (median GA, 29.4 weeks vs. 30.6 weeks, *p* = 0.001) and BW (median BW, 1225 g vs. 1400 g, *p* = 0.001) than the typical imaging finding (-) group, which was in agreement with similar research results (27). With regard to perinatal factors, the typical imaging finding (+) group had a higher sepsis rate (75.6 vs. 61.8%, *p* = 0.031) and higher RDS rate (84.6 vs. 73.0%, *p* = 0.044) than the typical imaging finding (-) group. In terms of treatment, the typical imaging finding (+) group was more likely to require postnatal glucocorticoid administration (80.8 vs. 65.7%, *P* = 0.015), long-term IMV (67.9 vs. 41.0%, *P* < 0.001) and more PRBC transfusions (46.2 vs. 30.9%, *P* = 0.019) than the typical imaging finding (-) group. After PSM, all 15 covariates were well balanced, and no significant differences were observed between the groups (*p* > 0.05) (Table 1).

### Primary and Secondary Outcomes

Outcomes are shown in Table 2. After PSM, the overall mortality in preterm infants with BPD was 20.7% (24 of 116), the proportion of severe BPD was 19.0% (22 of 116), and the rate of HOT at discharge was 60.3% (70 of 116). Patients with typical imaging findings demonstrated a remarkably higher mortality rate (29.3 vs. 12.1%, *p* = 0.022, OR 3.021, 95% CI 1.143–7.981). Infants with typical imaging findings differed significantly from the rest of the cohort in that they had a higher proportion of severe BPD (32.8 vs. 12.1%, *p* = 0.003, OR 4.669, 95% CI 1.723–12.652) and a higher rate of HOT at discharge (74.1 vs. 46.6%, *p* = 0.002, OR 3.291, 95% CI 1.506–7.195).

**Table 2:** Short-term outcomes and follow-up until 2 years of age of preterm infants in the typical imaging finding (±) groups after PSM.

	**Total (*n* = 116)**	**Typical imaging findings (−) (*n* = 58)**	**Typical imaging findings (+) (*n* = 58)**	**OR (95% CI)**	***P* value**
Severity of BPD					0.003
Mild BPD	68 (58.6)	43 (74.1)	25 (43.1)	Ref	
Moderate BPD	26 (22.4)	8 (13.8)	14 (24.1)	3.010 (1.109, 8.172)	
Severe BPD	22 (19.0)	7 (12.1)	19 (32.8)	4.669 (1.723,12.652)	
HOT at discharge	70 (60.3)	27 (46.6)	43 (74.1)	3.291 (1.506, 7.195)	0.002
Mortality^+^	24 (20.7)	7 (12.1)	17 (29.3)	3.021 (1.143, 7.981)	0.022
HS (days)	58 (41.2, 66.9)	56.5 (45.2, 65.0)	59 (33.8, 68.2)	1.000 (0.983, 1.017)	0.901
O2 (days)	49.0 (36.2, 64.0)	46 (38, 56.2)	56 (32.7, 68.2)	1.016 (0.997, 1.035)	0.263
		*N* = 51	*N* = 41		
Underweight^+^	7 (6.0)	4 (7.8)	3 (7.3)	0.928 (0.196, 4.400)	1.000
Stunting^+^	11 (9.5)	7 (13.7)	4 (9.8)	0.680 (0.184, 2.503)	0.795
Wheezing disorders^+^	8 (6.9)	3 (5.9)	5 (12.2)	2.222 (0.498, 9.911)	0.487
Clinical visits^+^ <5 times 5–10 times >10 times	53 (57.6) 21 (22.8) 18 (19.6)	33 (64.7) 10 (19.6) 8 (15.7)	20 (48.8) 11 (26.8) 10 (24.4)	Ref 1.815 (0.654, 5.037) 2.063 (0.698, 6.092)	0.301
Rehospitalization^+^	1 (0, 2)	1 (0, 2)	1 (0, 3)	1.094 (0.950, 1.259)	0.670

*Data are presented as medians (IQRs) or numbers (%). ^+^follow-up until 2 years of age. BPD, Bronchopulmonary dysplasia; Ref, Reference; HOT, Home oxygen therapy; HS, Hospital stay*.

No significant differences were observed between the two groups regarding secondary outcomes after matching. Overall, the study cohort showed a higher rate of stunting and underweight than the general population, but there was no significant difference between the two groups (*p* = 0.795 and 1.000, respectively). Comparisons of the duration of hospital stay and oxygen therapy as well as the incidence of wheezing disorders also revealed no significant differences between the groups (*p* = 0.901, 0.263 and 0.285, respectively). Moreover, the number of clinical visits and readmissions due to respiratory causes in the first two years were not significantly different between the matched groups (*p* = 0.301 and 0.670, respectively).

### Independent Factors of Mortality

We completed the collinearity analysis and found that the variance inflation factors (VIFs) of all variables were <10, indicating that there was no obvious multicollinearity between the variables. After multivariate binary logistic regression analysis, the following variables were finally selected (Table 3). The factors associated with increased mortality were typical imaging findings found within 7 days and typical imaging findings after 7 days and IMV ≥ 7 days (OR 7.794, *p* = 0.004; OR 4.533, *p* = 0.001; OR 2.399, *p* = 0.038; respectively). NEC could be regarded as a risk factor for mortality with marginal statistical significance (OR 4.720, *p* = 0.060), whereas postnatal glucocorticoid administration and PIH could be regarded as protective factors against mortality (OR 0.201, *p* < 0.001; OR 0.200, *p* = 0.041; respectively).

**Table 3:** Multivariate logistic regression analysis for mortality in preterm infants with BPD.

**Variables**		**OR (95% CI)**	***p* Value**
IMV ≥ 7 d	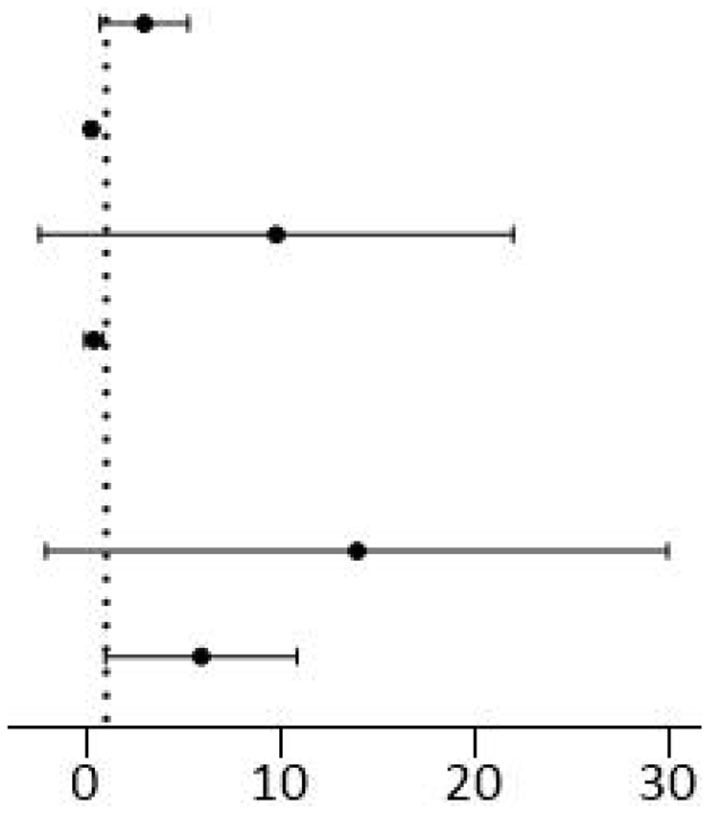	2.399 (1.049–5.484)	0.038
Postnatal glucocorticoids administration		0.201 (0.086–0.469)	<0.001
NEC		4.720 (0.939–23.713)	0.060
PIH		0.200 (0.043–0.939)	0.041
Typical imaging findings (−) Typical imaging findings ≤ 7 d Typical imaging findings > 7 d		Ref 7.794 (1.891–32.119) 4.533 (1.805–11.386)	0.001 0.004 0.001
			

*CI, confidence interval; NEC, necrotizing enterocolitis; PIH, pregnancy-induced hypertension; Ref, reference*.

## Discussion

BPD is a prevalent respiratory complication of preterm birth. Radiography plays a critical role in the clinical evaluation of BPD (13, 28, 29). Our data demonstrated that typical imaging findings were associated with the severity of BPD, a higher rate of mortality and HOT at discharge. In particular, the typical imaging findings in the early stage (within 7 days) were closely related to mortality.

In the original definition of BPD proposed by pediatric radiologist Northway in 1967 (30), abnormal chest radiographic findings were classified into four stages according to lung pathology. Serial chest radiographs indicated the evolution of BPD. As the survival rate of early preterm infants increased, the radiologic changes were not as classic as Northway proposed because of changed pathophysiology (31). However, neonatologists still attached great importance to imaging confirmation in BPD (32). Fibrotic opacities/cystic appearances in chest radiographs are recognized as the typical radiographic pattern of BPD (33); these appearances correlate with disease severity and are relatively easy to recognize. The typical imaging findings after 36 weeks may not have an impact on the diagnosis and severity of BPD, but they are closely related to mortality and have certain indications for clinical management and treatment.

The clinical ability to reliably predict the duration of respiratory support, let alone the outcomes of infants with BPD, is not yet sufficient. Exploring additional factors related to BPD is the key to improving its treatment and prognosis. Previous studies have reported that the findings of chest radiographs, such as interstitial shadows, fibrosis or bubbly/cystic appearances, could predict the incidence and severity of BPD (34) and have an association with respiratory outcomes. Some infants with moderate or severe BPD may have typical radiographic changes within the first 7 days of life (35). Ren et al. (36) revealed that infants with BPD-related imaging changes required a longer duration of IMV and parenteral nutrition and were more likely to require HOT. However, these studies contained insufficient clinical data and did not adjust for confounding factors. Most of them only paid attention to the chest X-ray appearance within 7 days or after 28 days. In addition, these studies did not analyze long-term prognosis, such as mortality. Therefore, further studies are needed to identify the association between typical imaging findings and outcomes.

The mortality rate of infants with BPD varies among different NICUs. Our study demonstrated that BPD infants with typical imaging findings had a higher probability of developing moderate to severe BPD, as well as a higher mortality rate, than those without typical imaging findings. The overall mortality rate in infants with BPD was 15.2% (39/256). The relatively high mortality rate of BPD is partially due to deficiencies in my country's medical conditions, which leads to delays in individualized and effective treatment.

The multivariate logistic regression analysis showed that typical imaging findings ≤ 7 days and typical imaging findings >7 days were risk factors for mortality in preterm infants with BPD (OR 7.794, *p* = 0.004; OR 4.533, *p* = 0.001, respectively). The significant associations we demonstrated between typical imaging findings and the severity of BPD and mortality are in accordance with previous findings. Infants who had undergone IMV ≥ 7 days had a 2.399 (95% CI 1.049–5.484) times higher risk of mortality than those who had undergone IMV <7 days (37), while postnatal glucocorticoid administration and a maternal PIH history were protective factors against mortality (OR 0.201, *p* < 0.001; OR 0.200, *p* = 0.041; respectively). This was consistent with other studies (38, 39). Interestingly, the risk factors for mortality in infants with BPD did not include GA or BW. Further work is needed to determine the clinical reason for this result.

Our study found that BPD infants with typical imaging findings were significantly more likely to require HOT at discharge (74.1 vs. 46.6%). The rates of HOT were influenced by age at discharge and discharge policies (40, 41) and varied widely among hospitals. The higher overall incidence of HOT at discharge in this study may be due to the heavy financial burden on the family and the desire for an earlier discharge to facilitate infant bonding at home. This may be why there was no significant difference in hospital stay or duration of oxygen supplementation between the two groups. The target oxygen saturation of HOT is higher than the oxygen-dependent standard, so the ratio of HOT in our study was higher than the ratio of moderate to severe BPD (42).

Our study indicated that typical imaging findings were associated with a higher rate of wheezing disorders (12.2 vs. 5.9%). However, no significant difference was noted between the groups (*p* = 0.285). If the follow-up period was longer, it would not be surprising to find a positive conclusion. For example, Arai et al. demonstrated that bubbly/cystic appearance on CXR after 28 days in infants with BPD was a potential risk factor for wheezing disorders at 3 years of age (43). Other studies showed that older children and young adult survivors of BPD had an increased risk of asthma (44–46).

Previous studies (47, 48) have shown that infants with BPD were more than twice as likely to be hospitalized during the first year of life as those without BPD. Our data demonstrated that the presence of typical imaging findings did not significantly increase the rate of rehospitalization or the frequency of clinical visits during the first two years after birth. Compared with infants without typical imaging findings, BPD infants with typical imaging findings did not have a higher proportion of underweight or stunting. This may be reflective of changes and improvements in clinical characteristics, postnatal treatment and admission criteria, which allows determination of and confidence in the clinical treatment of patients with typical imaging findings.

The exact etiologies of fibrotic and cystic changes are unclear and are likely to be associated with intrauterine inflammation. Intrauterine infection and CAM are believed to induce fetal lung inflammation, which could interfere with lung development, resulting in BPD. A meta-analysis confirmed that among preterm infants, exposure to CMA was associated with an increased risk of developing BPD (49). Hirata et al. (22) also reported an association between a cystic/bubbly pattern and intrauterine inflammation. The correlation of typical imaging findings with CAM is clouded by the complexity of fetal exposure to inflammation. Additional studies are needed to show the relationship between typical chest imaging findings and CAM.

Some limitations of the study are as follows: (1) this is a retrospective study, which inevitably leads to loss of follow-up and clinical data. A standardized follow-up program has not been perfected in our unit, especially 3 years ago or even earlier. Poor follow-up compliance was related to different geographical origins of enrolled infants and parents' reluctance to travel a long distance. We routinely asked about the condition of the infant after being discharged from the hospital by phone. Some information was not easy to obtain, there was a certain probability of bias. Nevertheless, we tried our best to verify relevant information and only presented reliable data. (2) This is a single-center study of Chinese premature infants. Our unit did not perform the oxygen reduction test, causing some infants to receive a longer time of supplemental oxygen (50). There are some differences in the incidence and severity of some complications from other international studies (51). (3) We used propensity score matching to balance some of the known confounding variables between the groups, making the outcomes more comparable and the conclusion more reliable and stable. However, it is inevitable to pay the cost of missing samples. (4) In addition, we only evaluated the role of typical imaging findings in preterm infants with BPD. Perhaps some infants with similar chest imaging findings would not even have BPD at all. In the later stage, we could work with the radiology department to conduct a larger population with a wider gestational age study.

Despite these limitations, this study provides a new perspective for understanding chest imaging in BPD. Typical chest imaging findings combined with medical history and clinical characteristics can provide powerful help for early diagnosis and treatment.

## Conclusion

This study provides important and fresh information that helps explain the potential harms that might be expected when typical imaging findings are noted in infants with BPD. In summary, our results demonstrated that typical imaging findings were significantly associated with the severity of BPD and a higher rate of mortality in this retrospective study. BPD infants with typical imaging findings also accounted for a higher proportion of infants requiring HOT at discharge.

To identify the influence of chest radiographic findings on long-term respiratory and neurodevelopmental outcomes, further prospective randomized controlled studies with larger samples and strict follow-up are needed.

## Data Availability Statement

The original contributions presented in the study are included in the article/supplementary material, further inquiries can be directed to the corresponding author.

## Ethics Statement

The studies involving human participants were reviewed and approved by the Institutional Review Board of Children's Hospital of Chongqing Medical University (2019-209). Written informed consent from the participants' legal guardian/next of kin was not required to participate in this study in accordance with the national legislation and the institutional requirements.

## Author Contributions

QR participated in the design of the study, collected clinical data, conducted statistical analyses, provided interpretation of data, drafted the initial manuscript, and reviewed the final manuscript as submitted. JW conceptualized and designed the study, provided interpretation of data, and revised the article for important intellectual content. YS conceptualized and supervised the design and execution of the study, reviewed analyses, and critically reviewed and revised the manuscript for important intellectual content. All authors contributed to the article and approved the final manuscript as submitted.

## Conflict of Interest

The authors declare that the research was conducted in the absence of any commercial or financial relationships that could be construed as a potential conflict of interest.

## Publisher's Note

All claims expressed in this article are solely those of the authors and do not necessarily represent those of their affiliated organizations, or those of the publisher, the editors and the reviewers. Any product that may be evaluated in this article, or claim that may be made by its manufacturer, is not guaranteed or endorsed by the publisher.

## Abbreviations

GA: Gestational age
BW: Birth weight
PIH: Pregnancy-induced hypertension
CAM: Chorioamnionitis
RDS: Respiratory distress syndrome
PDA: Patent ductus arteriosus
RSD: Repeated surfactant dose
IMV: Invasive mechanical ventilation
PRBC: Packed red blood cell
IVH: Intraventricular hemorrhage
NEC: Necrotizing enterocolitis
CI: Confidence interval
HOT: Home oxygen therapy
BPD: Bronchopulmonary dysplasia
ROP: Retinopathy of prematurity.
